# Supplementary material for: The genome sequence of an orbweaving spider, Gibbaranea gibbosa (Walckenaer, 1802)
Source: Wellcome Open Res. 2025 Feb 24;10:97. [Version 1] doi: 10.12688/wellcomeopenres.23751.1 (PMC11953616; doi:10.12688/wellcomeopenres.23751.1)
Supplement: Peer review history

# Reviewer report: Reviewer response for version 1

**DOI:** 10.21956/wellcomeopenres.26199.r119512

**Published:** 2025-03-28

**Authors:** Jessica E Garb

**Affiliations:** 1 University of Massachusetts Lowell,, Lowell, MA, USA

This data note reports a chromosome level genome for the spider *Gibbaranea gibbosa*, the only extant representative of this genus in Britain and Ireland.  The work follows the protocols of the Wellcome Sanger Trust Darwin Tree of Life project and appears thorough in terms of producing a high-quality genome assembly.  This is a nice contribution to the growing genomic resources for spiders.

 I have a few comments related to issues I am interested in and perhaps things for the authors to think about going forward.

  

1. It would be worth collecting at least one more individual, if not an additional male and female from the same locality as the specimens used for the genome sequencing to be deposited in a museum as a morphological voucher.  That may become important if there is a need to document some aspect of phenotype in relation to the genome. 
2. The use of mitochondrial genotyping of the sequenced specimen and comparing to the BOLD database makes sense for confirming the species identity, but the database is only as reliable as the original input data and how close the match is.  The authors could briefly comment on how good the match of their sequenced specimen’s barcode is to those that it matches in the database with this species name.  The paper only says the species was confirmed but not the level of similarity.
3. The paper states the genome will be annotated using available RNA-Seq data and presented through the Ensemble pipeline at the European Bioinformatics Institute.  It is unclear if the available RNA-Seq data will be from other spider species or from this species ( *Gibbaranea gibbosa*).  It would be ideal and seemingly not much more effort to include additional specimens from the same population for RNA-Seq for annotation.  While an annotation based on other spider species RNA/protein sequences may be reasonable and probably could yield good BUSCO scores, most likely it will miss out on finding and/or properly annotating the more interesting and rapidly evolving genes like those involved in venom and silk, which are what many people in the community would be interested in. 
4. The authors say that for the Pacbio HiFi sequencing that samples were required to have an average fragment size greater than 8kb and a total mass over 400 ng to proceed to the low input smrt bell prep kit 3.0 protocol.  But then as far as I can tell they never say how much DNA they were able to obtain from the single specimen and whether they definitely did use the low input protocol – it is implied they did.  I would like to know the amount of DNA obtained from their specimen before and after size selection.
5. Can the authors confirm whether or not any PCR was involved in generating the primary PacBio sequence data? That would be helpful for me to know.
6. Like one of the other reviewers, I’d also like to read a few more details on how the HiC data provides signal to infer which are the sex chromosomes. 

Are sufficient details of methods and materials provided to allow replication by others?

Yes

Is the rationale for creating the dataset(s) clearly described?

Yes

Are the datasets clearly presented in a useable and accessible format?

Yes

Are the protocols appropriate and is the work technically sound?

Yes

Reviewer Expertise:

Genomics, Evolutionary Biology, Spider Biology, Silk and Venom Protein evolution

I confirm that I have read this submission and believe that I have an appropriate level of expertise to confirm that it is of an acceptable scientific standard.

# Reviewer report: Reviewer response for version 1

**DOI:** 10.21956/wellcomeopenres.26199.r120108

**Published:** 2025-03-18

**Authors:** Guilherme Gainett

**Affiliations:** 1 Boston Children's Hospital F M Kirby Neurobiology Center, Boston, Massachusetts, USA

This paper provides a chromossome-level genome assembly for a orb-weaving spider, as part of the Darwin Tree of Life initiative. The genome sequencing was done with state-of-the-art PacBio long-read sequencing and Hi-C scaffolding.

 The description is clear and the genome quality measures are high, such as contiguity and completeness (ex: BUSCO).

 My one general concern is the HiC data, which does not seem to have resolution to show the chromosomes. It was not clear to me how much the HiC really contributed to the scaffolding of the genome. I think this point needs to be clarified in the text. 

 Besides this point, I also suggest providing additional details about how the sex chromosomes were inferred. There are also small nomenclature suggestions and minor comments in the **attached pdf**.

 This paper provides a great quality resource for the community.

Are sufficient details of methods and materials provided to allow replication by others?

Yes

Is the rationale for creating the dataset(s) clearly described?

Yes

Are the datasets clearly presented in a useable and accessible format?

Yes

Are the protocols appropriate and is the work technically sound?

Yes

Reviewer Expertise:

Arachnology; Developmental Biology; Genomics

I confirm that I have read this submission and believe that I have an appropriate level of expertise to confirm that it is of an acceptable scientific standard, however I have significant reservations, as outlined above.

# Reviewer report: Reviewer response for version 1

**DOI:** 10.21956/wellcomeopenres.26199.r119516

**Published:** 2025-03-06

**Authors:** Peng-Yu Jin

**Affiliations:** 1 University of the Chinese Academy of Sciences, Beijing, Beijing, China

This article is overall excellent, with a well-executed analytical approach and solid data. Notably, the use of PacBio and Hi-C sequencing on a single tiny individual provides valuable insights for studying other small invertebrates of similar size. Incorporating gene annotation files such as GFF would further enhance the study’s impact, particularly for research on spider traits like toxin, silk and behaviors. The Hi-C contact map of the chromosomes appears less distinct, as clear block patterns between different chromosomes are not readily visible. It would be helpful if the study could provide a more detailed Hi-C interaction map or discuss potential factors that might have influenced the resolution. Has a karyotype analysis been conducted for this species? If so, incorporating relevant information could further support the chromosome assembly.

Are sufficient details of methods and materials provided to allow replication by others?

Yes

Is the rationale for creating the dataset(s) clearly described?

Yes

Are the datasets clearly presented in a useable and accessible format?

Yes

Are the protocols appropriate and is the work technically sound?

Yes

Reviewer Expertise:

Single cell RNA sequencing, genome evolution, and nervous system  evolution of spider

I confirm that I have read this submission and believe that I have an appropriate level of expertise to confirm that it is of an acceptable scientific standard.
